# Pharmacokinetics of morphine in encephalopathic neonates treated with therapeutic hypothermia

**DOI:** 10.1371/journal.pone.0211910

**Published:** 2019-02-14

**Authors:** Laurent M. A. Favié, Floris Groenendaal, Marcel P. H. van den Broek, Carin M. A. Rademaker, Timo R. de Haan, Henrica L. M. van Straaten, Peter H. Dijk, Arno van Heijst, Jeroen Dudink, Koen P. Dijkman, Monique Rijken, Inge A. Zonnenberg, Filip Cools, Alexandra Zecic, Johanna H. van der Lee, Debbie H. G. M. Nuytemans, Frank van Bel, Toine C. G. Egberts, Alwin D. R. Huitema, on behalf of the PharmaCool study group

**Affiliations:** 1 Department of Clinical Pharmacy, University Medical Center Utrecht, Utrecht, the Netherlands; 2 Department of Neonatology, Wilhelmina Children's Hospital, University Medical Center Utrecht, Utrecht, the Netherlands; 3 Brain Center Rudolf Magnus, University Medical Center Utrecht, Utrecht, the Netherlands; 4 Department of Clinical Pharmacy, St. Antonius Hospital, Nieuwegein, the Netherlands; 5 Department of Neonatology, Emma Children’s Hospital, Academic Medical Center, Amsterdam, the Netherlands; 6 Department of Neonatology, Isala Clinics, Zwolle, the Netherlands; 7 Department of Neonatology, Groningen University Medical Centre, Groningen, the Netherlands; 8 Department of Neonatology, Radboud university medical center-Amalia Children’s Hospital, Nijmegen, the Netherlands; 9 Department of Pediatrics, Division of Neonatology, Erasmus Medical Centre-Sophia Children’s Hospital, Rotterdam, the Netherlands; 10 Department of Neonatology, Máxima Medical Center Veldhoven, Veldhoven, the Netherlands; 11 Department of Neonatology, Leiden University Medical Center, Leiden, the Netherlands; 12 Department of Neonatology, VU University Medical Center, Amsterdam, the Netherlands; 13 Department of Neonatology, UZ Brussel—Vrije Universiteit Brussel, Brussels, Belgium; 14 Department of Neonatology, University Hospital Gent, Gent, Belgium; 15 Paediatric Clinical Research Office, Emma Children’s Hospital, Academic Medical Center, University of Amsterdam, Amsterdam, The Netherlands; 16 Clinical Research Coordinator PharmaCool Study, Amsterdam, the Netherlands; 17 Department of Pharmacoepidemiology and Clinical Pharmacology, Faculty of Science, Utrecht University, Utrecht, the Netherlands; 18 Department of Pharmacy & Pharmacology, Netherlands Cancer Institute, Amsterdam, the Netherlands; University of Kentucky, UNITED STATES

## Abstract

**Objective:**

Morphine is a commonly used drug in encephalopathic neonates treated with therapeutic hypothermia after perinatal asphyxia. Pharmacokinetics and optimal dosing of morphine in this population are largely unknown. The objective of this study was to describe pharmacokinetics of morphine and its metabolites morphine-3-glucuronide and morphine-6-glucuronide in encephalopathic neonates treated with therapeutic hypothermia and to develop pharmacokinetics based dosing guidelines for this population.

**Study design:**

Term and near-term encephalopathic neonates treated with therapeutic hypothermia and receiving morphine were included in two multicenter cohort studies between 2008–2010 (SHIVER) and 2010–2014 (PharmaCool). Data were collected during hypothermia and rewarming, including blood samples for quantification of morphine and its metabolites. Parental informed consent was obtained for all participants.

**Results:**

244 patients (GA mean (sd) 39.8 (1.6) weeks, BW mean (sd) 3,428 (613) g, male 61.5%) were included. Morphine clearance was reduced under hypothermia (33.5°C) by 6.89%/°C (95% CI 5.37%/°C– 8.41%/°C, p<0.001) and metabolite clearance by 4.91%/°C (95% CI 3.53%/°C– 6.22%/°C, p<0.001) compared to normothermia (36.5°C). Simulations showed that a loading dose of 50 μg/kg followed by continuous infusion of 5 μg/kg/h resulted in morphine plasma concentrations in the desired range (between 10 and 40 μg/L) during hypothermia.

**Conclusions:**

Clearance of morphine and its metabolites in neonates is affected by therapeutic hypothermia. The regimen suggested by the simulations will be sufficient in the majority of patients. However, due to the large interpatient variability a higher dose might be necessary in individual patients to achieve the desired effect.

**Trial registration:**

www.trialregister.nl NTR2529.

## Introduction

Hypoxic-ischemic encephalopathy (HIE) following perinatal asphyxia is one of the leading causes of death and disability in term and near term neonates. Therapeutic hypothermia (TH, lowering the core temperature to 33–34° C for 72h) is an established neuroprotective strategy and has become standard of care for these patients in developed countries.[1,2] In the Netherlands, approximately 150–200 neonates receive this treatment annually using whole-body cooling.[3]

Morphine is a commonly used drug in hypothermic neonates to provide analgesia and sedation, and is considered an important drug since stress may reduce the neuroprotective effects of TH.[4] Morphine undergoes extensive hepatic metabolism and its predominant metabolite is morphine-3-glucuronide (M3G) which is non-sedative. The less abundant metabolite morphine-6-glucuronide (M6G) is pharmacologically active with similar or greater sedative and analgesic effects compared to the parent compound.[5,6] Both glucuronide metabolites are formed by UDP glucuronosyltransferase 2B7 (UGT2B7).[5] The UGT2B7 enzyme activity in neonates is less than 10% of that in adults, but increases rapidly during the first days after birth.[7,8] Both metabolites are eliminated through the kidneys.[5] At birth, renal function is underdeveloped compared to older children and adults. In the first few weeks of life, a steady increase in renal function can be seen.[8] Thus, maturation of kidney function might influence metabolite elimination.[8,9]

Hypothermia might influence numerous physiological processes involved in drug metabolism. Hypothermia reduces cardiac output and increases vascular resistance, which leads to decreased liver perfusion. Decreased liver perfusion might result in decreased drug clearance, especially in drugs with a high hepatic extraction ratio. Furthermore, the activity of liver enzymes such as cytochrome P450 and UGT27B can be declined during TH resulting in impaired clearance. Likewise, TH can decrease renal drug clearance by reducing kidney perfusion and subsequent glomerular filtration but also through changes in tubular secretion and reabsorption.[10–12] Additionally, pharmacokinetics (PK) of drugs administered to these neonates may be altered due to hypoxia-ischemia related multi-organ failure.[13,14] In recent years, studies have been conducted that investigated the PK of frequently used drugs in neonates undergoing TH. The findings of these studies have led to dose recommendations for several antibiotics and anticonvulsive drugs.[15–21]

Morphine PK in normothermic neonates has been investigated in several studies, mostly involving preterm and term neonates following major thoracic and abdominal surgery.[22] Neonates with a postnatal age (PNA) below 10 days had a markedly reduced morphine clearance compared to older children which has been attributed to impaired glucuronidation. This effect was independent of birth weight (BW) or gestational age (GA). Maintenance dose in this group was reduced by 50% compared to older children to achieve morphine plasma concentrations between 10 and 40 μg/L.[9] This dosing algorithm has been prospectively validated and body weight has been shown to accurately predict morphine clearance across the entire pediatric population.[23–25]

Morphine PK in neonates with HIE undergoing TH has only sparsely been investigated. Róka et al. (2008) found elevated morphine plasma concentrations in neonates treated with TH (N = 10) compared to non-asphyxiated normothermic controls (N = 6) with similar infusion rates and cumulative doses.[26] Frymoyer et al. (2016) developed a population PK model for morphine, M3G and M6G during TH using data from 20 neonates. They concluded that morphine clearance during TH was lower compared to previous studies in normothermic asphyxiated neonates and advised a loading dose of 50 μg/kg followed by 5 μg/kg continuous infusion.[27] Both studies did not include data during and after rewarming. Additional characterization of morphine PK using a larger dataset is imperative to guide clinicians in the application of this widely used and important drug in this critically ill population.

The objective of the present study was to describe the PK of morphine and its metabolites in neonates with HIE both during and after TH using nonlinear mixed effect modelling and to develop pharmacokinetics based dosing guidelines based on a large dataset obtained from two multicenter studies conducted in the Netherlands and Belgium.

## Patients and methods

### Setting, study design and study population

The open label prospective SHIVER study was performed in the tertiary neonatal intensive care units (NICU) of the University Medical Center Utrecht, Utrecht and Isala Clinics, Zwolle. The open label prospective PharmaCool study was conducted in twelve tertiary NICUs in the Netherlands and Belgium.[28] In both studies, term neonates undergoing TH for HIE were eligible for inclusion. According to national treatment protocol, neonates with a GA between 36.0 and 42.0 weeks were cooled within 6 hours after birth to a core temperature of 33.5°C (accepted range 33.0–34.0°C) for 72 hours. Thereafter, patients were slowly (0.4°C/hour) rewarmed to normothermia (36.5°C). After rewarming, body temperature was stabilized at 36.5°C for 24 hours.[3] Exclusion criteria were severe congenital malformations, encephalopathy due to other causes than perinatal asphyxia and the absence of central venous or arterial access for non-invasive blood sampling. From each included patient, written parental informed consent was obtained. Inclusion took place between 2008–2010 (SHIVER) and 2010–2014 (PharmaCool). In total, 339 patients were screened and 277 included. For the present study, neonates participating in either study and receiving intravenous morphine were selected. Data analysis was completed in 2018. The SHIVER study was approved by the Institutional Review Board (IRB) of the University Medical Center Utrecht (no. 08/404) and subsequently approved by the IRB of the Isala Clinics, Zwolle. The PharmaCool study was approved by the IRB of the Academic Medical Center Amsterdam (no. 10/255) and subsequently approved by the IRBs of the VU Medical Center Amsterdam, University Medical Center Utrecht, Leiden University Medical Center, Erasmus Medical Center Rotterdam, Maxima Medical Center Veldhoven, Maastricht University Medical Center, Radboud University Medical Center Nijmegen, Isala Clincs Zwolle, University Medical Center Groningen, University Hospital Gent and University Hospital Brussels.

### Morphine dosing and administration

In both studies, morphine was administrated as morphine hydrochloride according to local protocols and/or the attending physician’s discretion as an intravenous continuous infusion, often preceded by a loading dose. Morphine was generally started at the onset of TH or shortly before. Dose adjustments, including administration of any additional loading dose, were based on each patient’s clinical condition and were not influenced by the study protocol.

### Pharmacokinetic sampling and bioanalysis

From all patients, 1 ml blood samples were obtained from an indwelling catheter on four consecutive days, both during hypothermia and rewarming/normothermia. Sampling was scheduled at designated time points at 24 hours intervals. This limited sampling strategy was designed to minimize patient risk while still obtaining sufficient information to achieve the study objective. In the SHIVER study, residual material from blood samples taken for clinical care were available for some patients. Plasma concentrations of morphine, M3G and M6G were determined using liquid chromatography-tandem mass spectrometry (LC-MS/MS). The lower limit of quantification (LLQ) was 10 μg/L for morphine and M3G and 5 μg/L for M6G. The calibration curves were linear from 10 to 1000 μg/L for morphine, 10 to 600 μg/L for M3G and 5 to 200 μg/L for M6G. Between-run and within-run coefficients of variation were <5% for morphine and M3G and <8% for M6G. Samples were stored at -80°C until analyses at the Clinical Pharmaceutical and Toxicological Laboratory of the Department of Clinical Pharmacy of the University Medical Center Utrecht, the Netherlands.

### Population pharmacokinetic analysis

A population pharmacokinetic model was developed from morphine, M3G, and M6G concentration–time data using the nonlinear mixed effect modelling program NONMEM (version 7.3, Icon Development Solutions) with R (version 3.4.1), Xpose (version 4) for data visualization and Piraña for run management.[29] Morphine hydrochloride (molecular weight (MW) 321.8 g/mol) doses were converted to morphine base (MW 285.3 g/mol) and consecutively, all units of dose and concentration for morphine, M3G and M6G (MW 461.5 g/mol) were converted to μmol and μmol/L, respectively for the purpose of the pharmacokinetic analysis. BW was used as a descriptor for body size in our population and was related to pharmacokinetic parameters using allometric relationships. The exponent defining the relationship of BW and clearance (Cl) was fixed to 0.75 and the exponent defining the relationship of BW and volume of distribution (V) was fixed to 1. The fractions of morphine converted to the metabolite M3G and M6G in neonates under hypothermia were unknown (F_M3G_ and F_M6G_, respectively); therefore, parameters relative to F were estimated (e.g. Cl_M3G_/F_M3G_ and V_M3G_/F_M3G_). Based on previously published pharmacokinetic models of morphine in neonates[27,30], one- and two-compartment models for morphine and subsequent one-compartment models for both metabolites were tested as a structural model. Morphine and metabolite data were fitted simultaneously.

To study the effect of hypothermia on pharmacokinetics, a dynamic model of temperature over time was included, which allowed prediction of the actual body temperature at each moment of sampling. For all patients, the reported start and end times of TH were used to determine the period of TH treatment. Body temperature during hypothermia was set at 33.5°C, with consecutive rewarming at 0.4°C/hour (i.e. rewarming time 7.5h) until 36.5°C after which body temperature was set to 36.5°C for the remainder of the study time. Calculated body temperature for each plasma sample was subsequently included in the PK model.

As renal function may be an important determinant for metabolite clearance and given that renal function cannot be estimated from a single serum creatinine (SCr) measurement in neonates, a model for SCr was developed using daily SCr values taken for clinical care from all patients. In this model, the elimination rate of SCr was used as a surrogate marker for renal function. PNA and GA were tested as covariates on both morphine and metabolite clearance. Inclusion of covariates was guided by effect size, biological plausibility and statistical significance (using the likelihood ratio test which assesses the difference in the NONMEM objective function value (OFV), which is equal to minus twice the log likelihood, with a p-value of <0.05 as cut-off for significance).

Interindividual variability (IIV) was modelled using a proportional model and tested on all parameters. Covariance between IIV components was included based on physiological plausibility and graphical exploration. A proportional error model was used to model residual unexplained variability. For each compound, separate error models were used. Parameter precision was assessed with sampling importance resampling (SIR).[31] Internal validation of the final model was evaluated by computing the normalized prediction distribution errors (NPDE, 1000 simulations).[32] Both graphical (e.g. goodness-of-fit plots, visual predictive check) and statistical model evaluation procedures were used to assess model adequacy.

### Dosing regimen development

Simulations were conducted to test four different dosing regimens using the parameter estimates from the final pharmacokinetic model. To create the simulation dataset, the patient characteristics of each neonate included in this study were replicated five times. Morphine loading dose was simulated at PNA 4 hours, immediately followed by continuous infusion. The following dosing regimens were evaluated, based on the current clinical practice: 1. loading dose of 50 μg/kg followed by continuous infusion of 5 μg/kg/h; 2. loading dose of 50 μg/kg, continuous infusion of 10 μg/kg/h; 3. loading dose of 100 μg/kg, continuous infusion of 5 μg/kg/h; 4. loading dose of 100 μg/kg, continuous infusion of 10 μg/kg/h. The dynamic temperature model was used to introduce TH. For each neonate in the simulation dataset, TH (body temperature of 33.5°C for 72 hours) was simulated to start at PNA 5 hours, after which rewarming commenced at 0.4°C/hour. After rewarming, body temperature was fixed to 36.5°C for the remainder of the simulations. Hourly plasma concentrations were predicted until PNA 120 hours. Morphine plasma concentrations between 10 and 40 μg/L were considered effective and safe.

## Results

### Patient characteristics

For 244 neonates morphine dosing information and at least one morphine plasma concentration was available for analysis (Table 1). In general, loading doses between 50 and 100 μg/kg were given, followed by continuous infusion with doses varying between 5 and 25 μg/kg/h.

**Table 1:** Patient characteristics.

Parameter	Patients (N = 244)
Gestational age; wk, mean ± sd	39.8 ± 1.6
Birth weight; g, mean ± sd	3,428 ± 613
*Birth weight ≤ 2500 g; n (%)*	16 (6.6%)
Male; n (%)	150 (61.5%)
pH*; median (IQR)	6.96 (6.80–7.09)
Base Excess*; mmol/L, median (IQR)	-17 (-12.0 –-21.9)
Lactate*; mmol/L, median (IQR)	13.6 (9.0–18.2)
Thompson score^#^; median (IQR)	9.5 (8.0–12.0)
aEEG on admission^#^	
*Continuous normal voltage; n (%)*	30 (12.3%)
*Discontinuous normal voltage; n (%)* *of whom < 5 μV; n (%)*	102 (41.8%) 35 (14.3%)
*Burst suppression; n (%)*	58 (23.8%)
*Continuous low voltage; n (%)*	10 (4.1%)
*Flat trace; n (%)*	27 (11.1%)
*Unknown; n (%)*	17 (7.0%)
Mortality; n (%)	58 (23.8%)

sd = standard deviation, IQR = interquartile range

*Value measured in umbilical cord blood or, if unavailable, from arterial or venous blood within 1h after birth

^#^Encephalopathy was characterized by a Thompson score of >7 1h after birth or an abnormal aEEG on admission to a level III NICU

**Fig 1:**
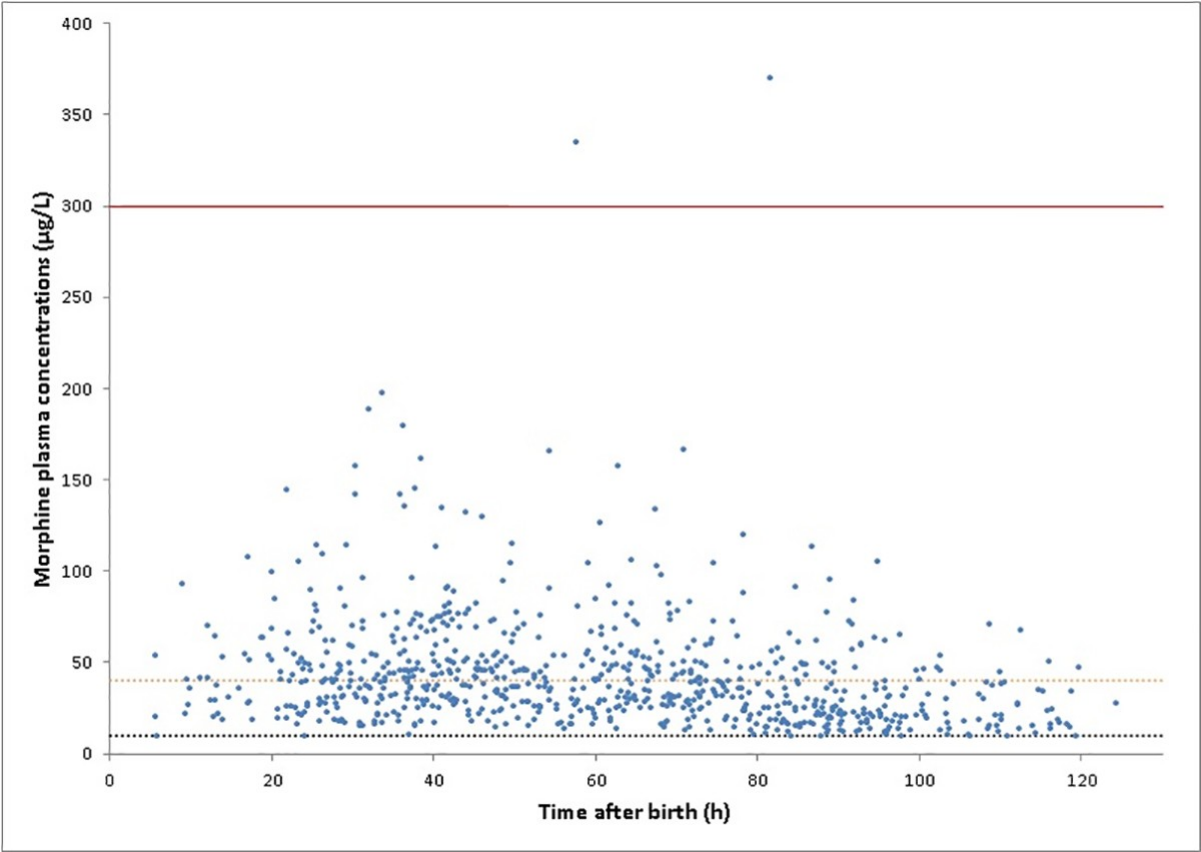
Observed morphine plasma concentrations (μg/L). Dotted lines indicate the proposed therapeutic window of 10–40 μg/L; solid line indicates the potentially toxic limit of 300 μg/L.

A total of 853 blood samples were analyzed (median 4 samples per patient, range 1–11). Samples with measurements below LLQ for all compounds (n = 23) were excluded from further analyses, leaving 830 viable samples. Of these, 550 (66.3%) were drawn during the hypothermic phase. For 18 patients (7.4%), only one sample was available. Plasma concentrations for morphine varied between 10.0 and 371.2 μg/L (Fig 1); for M3G between 11.0 and 930.6 μg/L and for M6G between 5.1 and 211.2 μg/L (S1 Fig). In one patient (0.41%), morphine plasma concentrations exceeding 300 μg/L were reached.

### Population pharmacokinetic analysis

A one-compartment model for morphine and subsequent one-compartment models for both metabolites provided the best fit for the data. Pharmacokinetic parameter estimates of the final model are shown in Table 2.

**Table 2:** Final model pharmacokinetic parameter estimates and SIR results.

	Morphine		M3G§		M6G§	
*Parameter*	Estimate	SIR* 95% CI	Estimate	SIR* 95% CI	Estimate	SIR* 95% CI
Cl, l/h^#^	0.899	0.797–0.985	0.456	0.424–0.492	1.73	1.61–1.87
V, l^#^	8.88	7.87–9.92	0.264	0.089–0.384	4.53	3.64–5.39
PNA on Cl; %/h	0.420	0.297–0.582	NA	NA	NA	NA
GA on Cl; %/d	1.66	1.30–1.94	NA	NA	NA	NA
TEMP on Cl; %/°C	6.89	5.37–8.41	4.91^$^	3.53–6.22^$^	4.91^$^	3.53–6.22^$^
*Interindividual variability*						
Cl, variance (rsd)	0.224 (47.3%)	0.185–0.276	0.291^$^ (53.9%)	0.240–0.356^$^	0.291^$^ (53.9%)	0.240–0.356^$^
V, variance (rsd)	0.464 (68.1%)	0.364–0.602	NA	NA	NA	NA
*Covariance interindividual variability Cl*_*morphine*_*/Cl*_*metabolites*_						
Covariance (correlation coefficient)	0.117 (46.0%)	0.0799–0.161				
*Residual variability*						
Proportional, variance (rsd)	0.0498 (22.3%)	0.0437–0.0574	0.0914 (30.2%)	0.0798–0.105	0.101 (31.8%)	0.0888–0.115

Final model

Cl_MORPHINE_ = 0.899 x (BW/3.5)^0.75^ x (1 + 0.0042 x PNA) x (1 + 0.0166 x (GA-280)) x (1 + 0.0689 x (TEMP-36.5))

V_MORPHINE_ = 8.88 x (BW/3.5)^1^

Cl_M3G_/F_M3G_ = 0.456 x (BW/3.5)^0.75^ x (1 + 0.0491 x (TEMP-36.5)

V_M3G_/F_M3G_ = 0.264 x (BW/3.5)^1^

Cl_M6G_/F_M6G_ = 1.73 x (BW/3.5)^0.75^ x (1 + 0.0491 x (TEMP-36.5)

V_M6G_/F_M6G_ = 4.53 x (BW/3.5)^1^

^§^All metabolite estimates are relative to formation fraction F_M3G_ and F_M6G_, resp.

*Ten iterations; no. of samples 1000,1000,1000,1000,1000,1000,2000,2000,2000,2000; no. of resamples 200,200,400,400,500,500,1000,1000,1000,1000

^#^Estimates for neonate with BW 3.5 kg, GA 280 days, PNA 0h and TEMP 36.5°C

^$^Single estimate for both metabolites

V = volume of distribution, Cl = clearance, PNA = postnatal age, GA = gestational age, TEMP = body temperature, M3G = morphine-3-glucuronide, M6G = morphine-6-glucuronide, SIR = sampling importance resampling, BW = birth weight, NA = not applicable, rsd = relative standard deviation

Introduction of a peripheral compartment for morphine resulted in an unstable model with unrealistic intercompartmental clearance. GA and PNA were identified as covariates on morphine clearance (GA: p<0.001, PNA: p<0.001), but not on metabolite clearance. Morphine clearance was increased by 50.4% at PNA 5 days, compared to birth (increase of 0.42%/h, 95%CI 0.297%/h– 0.582%/h); at birth, morphine clearance in a neonate with GA 36 weeks was 46% lower compared to GA 40 weeks, while clearance in a neonate with GA 42 weeks is 23% higher (difference of 1.66%/d, 95%CI 1.30%– 1.94%). The elimination rate of SCr was introduced as a covariate on the clearance of the metabolites as a measure of renal function. The influence of this covariate was non-significant and therefore excluded from the final model.

Subsequently, the dynamic model of temperature over time was included as covariate on Cl. The influence of body temperature on clearance was separated into an effect on Cl_MORPHINE_ (a combination of hepatic and renal clearance) and CL_METABOLITES_ (renal clearance). As the effect of body temperature on M3G and M6G clearance were similar and separate effects for both metabolites did not improve model performance, this was estimated as a single effect in the final model. Morphine clearance during hypothermia was decreased by 20.7% (p<0.001, 6.89%/°C, 95% CI 5.37%/°C– 8.41%/°C) compared to normothermia. Metabolite clearance during hypothermia was decreased by 14.7% (p<0.001, 4.91%/°C, 95% CI 3.53%/°C– 6.22%/°C).

**Fig 2:**
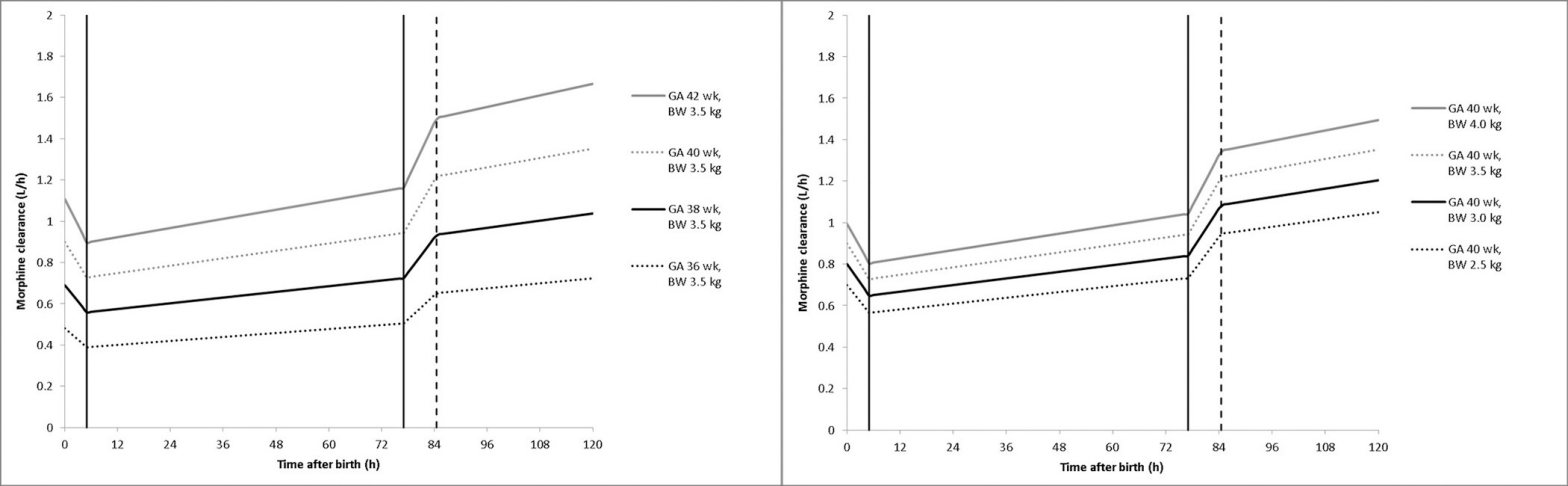
**Average predicted morphine clearance over time before, during and after TH for neonates with BW 3.5 and GA 36, 38, 40 and 42 weeks, respectively (left) and for neonates with GA 40 weeks and BW 2.5, 3.0, 3.5 and 4.0 kg, respectively (right).** Solid vertical lines represent the start and end of TH (33.5°C) simulated between 5h and 77h after birth; dashed vertical line indicates the return to normothermia (36.5°C) with rewarming simulated at. 0.4°C/h; TH = therapeutic hypothermia, BW = birth weight GA = gestational age.

The influence of BW, GA, PNA and temperature on the average morphine clearance is predicted by the final model are depicted in Fig 2.

Model evaluation demonstrated that the final model was adequate in describing the data. Goodness-of-fit plots of observed versus population and individual predicted concentrations showed no systematic deviation and the weighted residuals were homogeneously scattered for both parent and metabolites (S2–S4 Figs). NPDE plots for morphine, M3G and M6G indicate that the NPDE follows the normal distribution and that the model does not contain major bias (S5–S7 Figs).

### Dosing regimen

**Fig 3:**
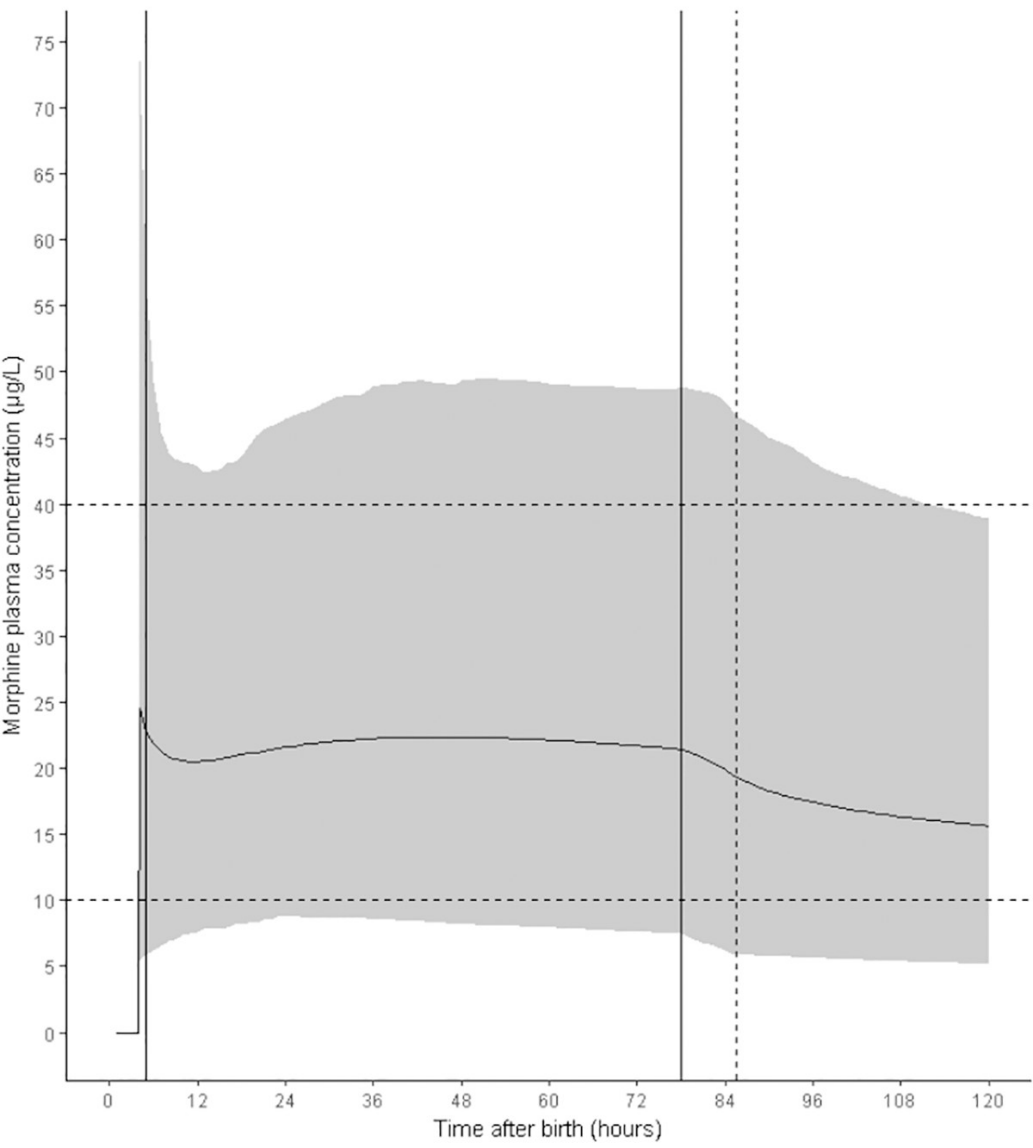
Simulated morphine plasma concentrations of the proposed dosing regimen of 5 μg/kg/h after loading dose of 50 μg/kg. Solid line indicates the mean morphine plasma concentration; gray area represents the 95% prediction interval. Dotted horizontal lines indicate the proposed therapeutic window of 10–40 μg/L. Solid vertical lines indicate the start and end of TH (33.5°C) simulated between 5h and 77h after birth; dashed vertical line indicates the return to normothermia (36.5°C) with rewarming simulated at 0.4°C/h; TH = therapeutic hypothermia.

Morphine plasma concentrations after various dosing regimens were predicted using a simulation dataset of 1220 patients and the final PK parameter estimates. In all simulations, morphine clearance was markedly influenced by PNA and TH. Immediately after rewarming, average morphine clearance was increased by 63.4% compared to clearance at the start of TH. Of this increase, 29.6% could be attributed to an effect of PNA. A maintenance dose of 5 μg/kg/h preceded by a loading dose of 50 μg/kg resulted in plasma concentrations between 10 and 40 μg/L at PNA 12h in 88.2% of patients, while 7.8% of patients were below 10 μg/L and 4.0% above 40 μg/L. At PNA 48h, morphine plasma concentration exceeded 40 μg/L in 6.8% of patients. As clearance is not constant but increased over time, no steady state in morphine plasma concentration was reached in the first five days of life. At PNA 77 hours, TH was stopped resulting in an additional increase in clearance and drop in plasma concentration (Fig 3). Plasma concentrations for both metabolites accumulated during TH but reached steady state once clearance increased under normothermic conditions (S8 Fig). Morphine plasma concentrations for the other simulated dosing regimens are included as a supplement (S9 Fig).

## Discussion

This study combining data from two large multicenter studies shows that clearance of both morphine and its metabolites is reduced during hypothermia in neonates with HIE compared to normothermia. Furthermore, the impact of BW, GA and PNA on the PK of morphine and its metabolites has been quantified. Reduction in clearance during TH is most likely caused by a decrease in perfusion of the liver and kidneys. Additionally, an effect of TH on activity of UGT2B7, the enzyme responsible for metabolizing morphine into M3G and M6G, might explain why morphine clearance is more strongly affected than metabolite clearance.

Although a therapeutic window for morphine plasma concentrations has not been firmly established, especially in neonates undergoing TH, the best available evidence suggests a preferred range between 10 and 40 μg/L, while levels above 300 μg/L have been associated with respiratory depression and prolonged mechanical ventilation.[25,33–38] Based on the simulations performed in this study, a starting dose of 50 μg/kg followed by 5 μg/kg/h is recommended to achieve morphine plasma concentrations between 10 and 40 μg/L, although the large interpatient variability (47.3% for Cl and 68.1% for V, Table 2) might lead to higher (>40 μg/l) plasma concentrations in individual patients. Contrarily, a higher morphine dose may be needed in some patients to ensure effective treatment. Clinicians should not be reluctant to increase the maintenance dose if the starting dose proves inadequate for a patient’s clinical condition both during and, if morphine is not stopped simultaneously with TH, after hypothermia.

The current practice rarely leads to plasma concentrations exceeding the potentially toxic upper limit of 300 μg/L, but may lead to unnecessary high morphine exposure. Neonatal opioid use has been associated with impaired cognitive and behavioral development in animal studies.[39] Long term follow-up studies in humans suggest a possible negative effect in early childhood that does not persist later in life.[40–43] Conversely, adequate management of pain and discomfort is needed to improve recovery, to ensure the effectiveness of TH and to prevent adverse physiological responses such as changes in intrathoracic or arterial pressure and vasoconstriction of vital organs.[4,38,39]

Morphine PK was best described using a one-compartment model for morphine and subsequent one-compartment models for each of the glucuronide metabolites. Previously, PK of the parent compound has been adequately described using both one-compartment[44,45] and two-compartment models.[9,23,27] A recently published meta-model combining data obtained in neonates and older children from five separate studies proposed a one-compartment model for morphine PK.[30] Metabolite PK was adequately described using one-compartment models for each metabolite in all studies.[9,23,27,44] Parameter estimates from this model extrapolated to a neonate with GA 40 weeks and BW 3.5 kg result in a higher morphine clearance (1.54 l/h) and a lower volume of distribution (5.25 l) compared to our findings.[30] This might be explained by the fact that our patient population consisted of critically ill term neonates admitted to a NICU. The meta-model incorporated data form both term and preterm neonates and from older children and adults as well. In all included studies, morphine was administered for post-operative pain. Morphine parameter estimates reported in a small dataset by Frymoyer et al. in the same population are in accordance with our findings, despite the differences in the underlying PK model (Cl 0.765 l/h, V 8.02 l).[27] Also, the impact of TH on morphine clearance in our study is similar to the effect found by Róka et al., who compared hypothermic neonates to non-asphyxiated normothermic controls using a non-parametric approach (Cl 0.69 l/h vs 0.89 l/h, decrease of 22.5%).[26]

GA was identified as a significant covariate on morphine clearance despite the relatively narrow range of GA (36–42 weeks) in this population. Previous reports investigating the PK of other drugs in the PharmaCool study population have reported similar effects.[18–20] In the present study, this might be explained by a lower baseline UGT2B7 activity in neonates with a lower GA. However, due to the large interpatient variability, this finding did not translate into a dosing advise differentiated by GA. For each of the situations presented in Fig 2, the simulated dosing regimen yields average morphine plasma concentrations between 10 and 40 μg/L. The majority of patients included in this study had a GA between 38 and 41 weeks (202/244, 82.8%); a lower mg/kg dose for neonates with a GA of 36 and 37 weeks only marginally improved the fraction of patients within the therapeutic window at PNA 12 hours. Furthermore, a differentiated dosing regimen based on GA within this relatively small subpopulation of NICU patients is deemed undesirable as this will be error-prone. Therefore, the proposed dosing regimen is advised for all neonates treated with TH after HIE. Currently, TH is explored in preterm neonates (GA 34–35 weeks) as well.[46] Extrapolation of our results to these neonates should be done with caution due to the absence of this patient population in the current dataset.

Our data show an increase in morphine clearance during the first five days after birth. This effect could be identified independently of the effect of body temperature. The increase of clearance over time can be attributed to maturation of UGT2B7. Maturation of this enzyme in normothermic neonates has been described for morphine but also for other drugs predominantly glucuronidated by UGT2B7.[9,47–50] Recovery of organ function after perinatal asphyxia might also play a role in this observed increase in clearance during the first days of life. During asphyxia, the liver is deprived of oxygen, resulting in hepatocyte damage. Alanine aminotransferase (ALAT) and aspartate aminotransferase (ASAT) levels are commonly used to indicate hepatocyte damage and both are frequently elevated in neonates with HIE. Peak levels are reached within 72 hours and normalize within the first two weeks of life, indicating recovery of liver function in this timeframe.[51–53] Unfortunately, ALAT and ASAT are poor predictors for hepatic drug metabolism and cannot be reliably used to predict hepatic clearance. Therefore, it was not possible to distinguish between enzyme maturation and recovery of liver damage.

Introducing temperature as a continuous covariate allowed for a more precise estimate of the effect of body temperature on the pharmacokinetics of morphine and its metabolites since also samples during rewarming were available. Additionally, including temperature as a dichotomous covariate proved to be a less adequate fit for the data. Some assumptions had to be made for this dynamic model. Firstly, we assumed that the average body temperature of each neonate during TH was 33.5°C and that possible fluctuations between 33.0 and 34.0°C would have a negligible effect. Body temperature during TH was therefore fixed to 33.5°C. Secondly, we assumed that rewarming for each neonate occurred according to national protocol at 0.4°C/hour. In clinical practice, rewarming is sometimes slowed or halted if seizures occur during rewarming. As this information was not available in our dataset, rewarming for each neonate was set at 0.4°C/hour. Thirdly, as body temperature after TH is stabilized at 36.5°C for 24 hours, we opted for a fixed body temperature of 36.5°C for each neonate after rewarming. As morphine is primarily administrated to prevent stress during hypothermia and is often stopped with or shortly after TH, we believe that this assumption is an accurate representation of clinical practice. Lastly, we assume a linear effect between body temperature and clearance and therefore report and effect per°C. Although we have no evidence for non-linearity, the limited sampling strategy was insufficient to exclude this. However, in event of non-linearity, the reported effect is an average effect per°C between 33.5 and 36.5°C and does not alter the overall effect of TH on clearance.

SCr is a specific marker for renal function and is widely used in adults to predict reduced clearance of renally excreted drugs. In neonates, SCr levels in the first few days of life are confounded by maternal SCr levels due to maternal transfer. We considered a dynamic model of SCr over time a better predictor of changes in renal function. No relationship between serum creatinine and renal clearance of the metabolites could be identified. Additionally, increased metabolite clearance over time in the first five days of life could not be observed. This effect was found in the same population for amoxicillin and benzylpenicillin, drugs that are predominantly excreted renally in unmetabolized form.[18,20] Data collection up to five days after birth might have been too short to detect maturation of renal function since steady state M3G and M6G plasma concentrations are not reached during hypothermia (S8 Fig). Also, as maturation of renal function in the first few days after birth occurs simultaneously with TH, the effect of maturation on metabolite clearance might not be distinguishable from the effect of hypothermia.

Pharmacodynamics (PD) end points of morphine were not incorporated in the final model. Although the COMFORT-B score, as indicator for pain and stress, was routinely recorded in this population, the timing of this score in relation to morphine dosing was often unclear. Pain expression in hypothermic neonates differs from normothermic neonates, making it uncertain whether the COMFORT-B score is suitable for treatment evaluation in this population.[38,54,55] Additionally, this scale has not been developed to distinguish between adequate treatment effect (eg. adequate sedation) and supratherapeutic effects (eg. oversedation).

Strengths of this study are the large number of included patients recruited from twelve tertiary NICUs in two countries, making this study population representative for all neonates treated with TH after HIE. The dosing regimen advised in this study corresponds with the dosing advise postulated by Frymoyer et al.[27] Reconstructing the full profile of hypothermia and rewarming enabled us to accurately assess the influence of body temperature on clearance. In all participating centers, TH is applied using a uniform cooling device and with a joint treatment protocol, thereby decreasing the chance of treatment variation.[3]

Limitations are the lack of PD end points associated with morphine. Clinicians are insufficiently supported by robust tools to facilitate morphine dose adjustments. Development and validation of such an instrument should be the focus of future research. Subsequently, this tool could be used to prospectively validate our dosing regimen. Ideally, future PD studies will also incorporate M6G plasma concentrations for determining exposure-response as the contribution of this active metabolite to the effects attributed to morphine in neonates are largely unknown.

For ethical reasons, it was not possible to answer this research question using a prospective randomized controlled trial comparing hypothermic with non-hypothermic patients. Also, comparison to an adequate historical control group is not feasible as morphine nor metabolite plasma concentrations are available from before 2008.

## Conclusion

Clearance of morphine and its metabolites is reduced in neonates treated with TH for HIE. Even though the current clinical practice only very rarely leads to morphine plasma concentrations exceeding 300 μg/L, a relatively low starting dose of 50 μg/kg followed by continuous infusion of 5 μg/kg/h is recommended in critically ill neonates treated with TH for HIE. However, due to the large interpatient variability, the uncertainty regarding the supposed therapeutic window and the undesirable effect of discomfort in this population, a higher maintenance dose may be required if the starting dose proves inadequate for the clinical condition of the individual patient.

## Supporting information

S1 Fig **observed plasma concentrations for M3G (left) and M3G (right).** M3G = morphine-3-glucuronde, M6G = morphine-6-glucuronide.
(TIF)

S2 Fig Morphine goodness-of-fit plots. A = observed vs population predicted plasma concentrations; B = observed vs individual predicted plasma concentrations; C = population conditional weighted residuals vs population predicted plasma concentrations; D = population conditional weighted residuals vs time after birth; solid line indicates the linear regression line.
(TIF)

S3 Fig M3G goodness-of-fit plots. A = observed vs population predicted plasma concentrations; B = observed vs individual predicted plasma concentrations; C = population conditional weighted residuals vs population predicted plasma concentrations; D = population conditional weighted residuals vs time after birth; M3G = morphine-3-glucuronide; solid line indicates the linear regression line.
(TIF)

S4 Fig M6G goodness-of-fit plots. A = observed vs population predicted plasma concentrations; B = observed vs individual predicted plasma concentrations; C = population conditional weighted residuals vs population predicted plasma concentrations; D = population conditional weighted residuals vs time after birth; M6G = morphine-6-glucuronide; solid line indicates the linear regression line.
(TIF)

S5 Fig Normalized prediction distribution errors (NPDEs) of the final pharmacokinetic model for morphine. A = kernel density plot of NPDE with a normal, Gaussian distribution overlaid for comparative purposes; B = Q-Q plot of theoretical quantiles vs sample quantiles; C = NPDE vs Time; D = NPDE vs predicted plasma concentrations; solid lines in figures C and D represent the observed median, 5^th^ and 95^th^ percentiles, red box represent the predicted 90% confidence interval around the median, blue boxes represent the predicted 90% confidence intervals around the 5^th^ and 95^th^ percentiles.
(TIF)

S6 Fig Normalized prediction distribution errors (NPDEs) of the final pharmacokinetic model for M3G. A = kernel density plot of NPDE with a normal, Gaussian distribution overlaid for comparative purposes; B = Q-Q plot of theoretical quantiles vs sample quantiles; C = NPDE vs Time; D = NPDE vs predicted plasma concentrations; M3G = morphine-3-glucuronide; solid lines in figures C and D represent the observed median, 5^th^ and 95^th^ percentiles, red box represent the predicted 90% confidence interval around the median, blue boxes represent the predicted 90% confidence intervals around the 5^th^ and 95^th^ percentiles.
(TIF)

S7 Fig Normalized prediction distribution errors (NPDEs) of the final pharmacokinetic model for M6G. A = kernel density plot of NPDE with a normal, Gaussian distribution overlaid for comparative purposes; B = Q-Q plot of theoretical quantiles vs sample quantiles; C = NPDE vs Time; D = NPDE vs predicted plasma concentrations; M6G = morphine-6-glucuronide; solid lines in figures C and D represent the observed median, 5^th^ and 95^th^ percentiles, red box represent the predicted 90% confidence interval around the median, blue boxes represent the predicted 90% confidence intervals around the 5^th^ and 95^th^ percentiles.
(TIF)

S8 Fig **simulated plasma concentration time profiles for M3G (left) and M6G (right) of the proposed morphine dosing regimen of 5** μ**g/kg/h after loading dose of 50** μ**g/kg.** Solid line indicates the mean plasma concentration; gray area represents the 95% prediction interval. M3G = morphine-3-glucuronde, M6G = morphine-6-glucuronide.
(TIF)

S9 Fig **Simulated morphine plasma concentrations of the dosing regimens of 10** μ**g/kg/h after loading dose of 50** μ**g/kg (left), 5** μ**g/kg/h after loading dose of 100** μ**g/kg (center) and 10** μ**g/kg/h after loading dose of 100** μ**g/kg (right).** Solid line indicates the mean morphine plasma concentration; gray area represents the 95% prediction interval. Dotted horizontal lines indicate the proposed therapeutic window of 10–40 μg/L. Solid vertical lines indicate the start and end of TH (33.5°C) simulated between 5h and 77h after birth; dashed vertical line indicates the return to normothermia (36.5°C) with rewarming simulated at 0.4°C/h; TH = therapeutic hypothermia.
(TIF)
