# Supplementary figures and images for: Pharmacokinetics of morphine in encephalopathic neonates treated with therapeutic hypothermia
Source: PLoS One. 2019 Feb 14;14(2):e0211910. doi: 10.1371/journal.pone.0211910 (PMC6375702; doi:10.1371/journal.pone.0211910)

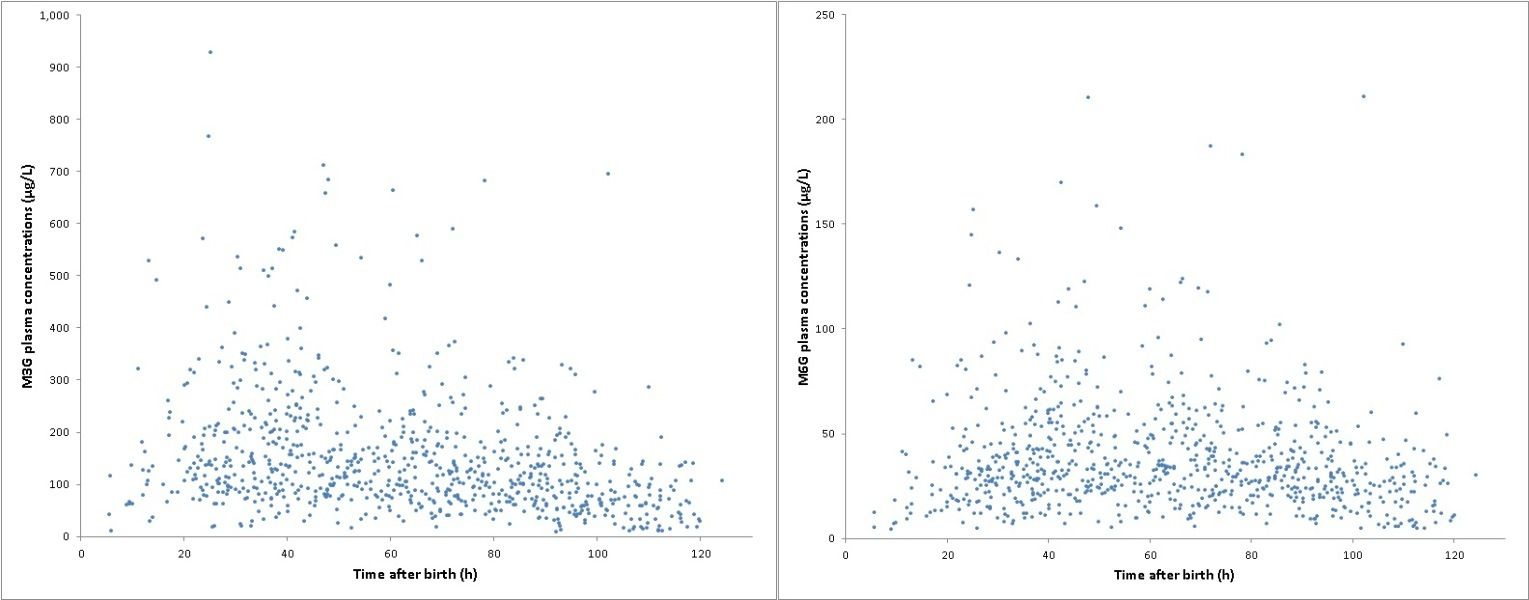

Supplement: S1 Fig — observed plasma concentrations for M3G (left) and M3G (right). M3G = morphine-3-glucuronde, M6G = morphine-6-glucuronide. (TIF) [file pone.0211910.s001.tif]

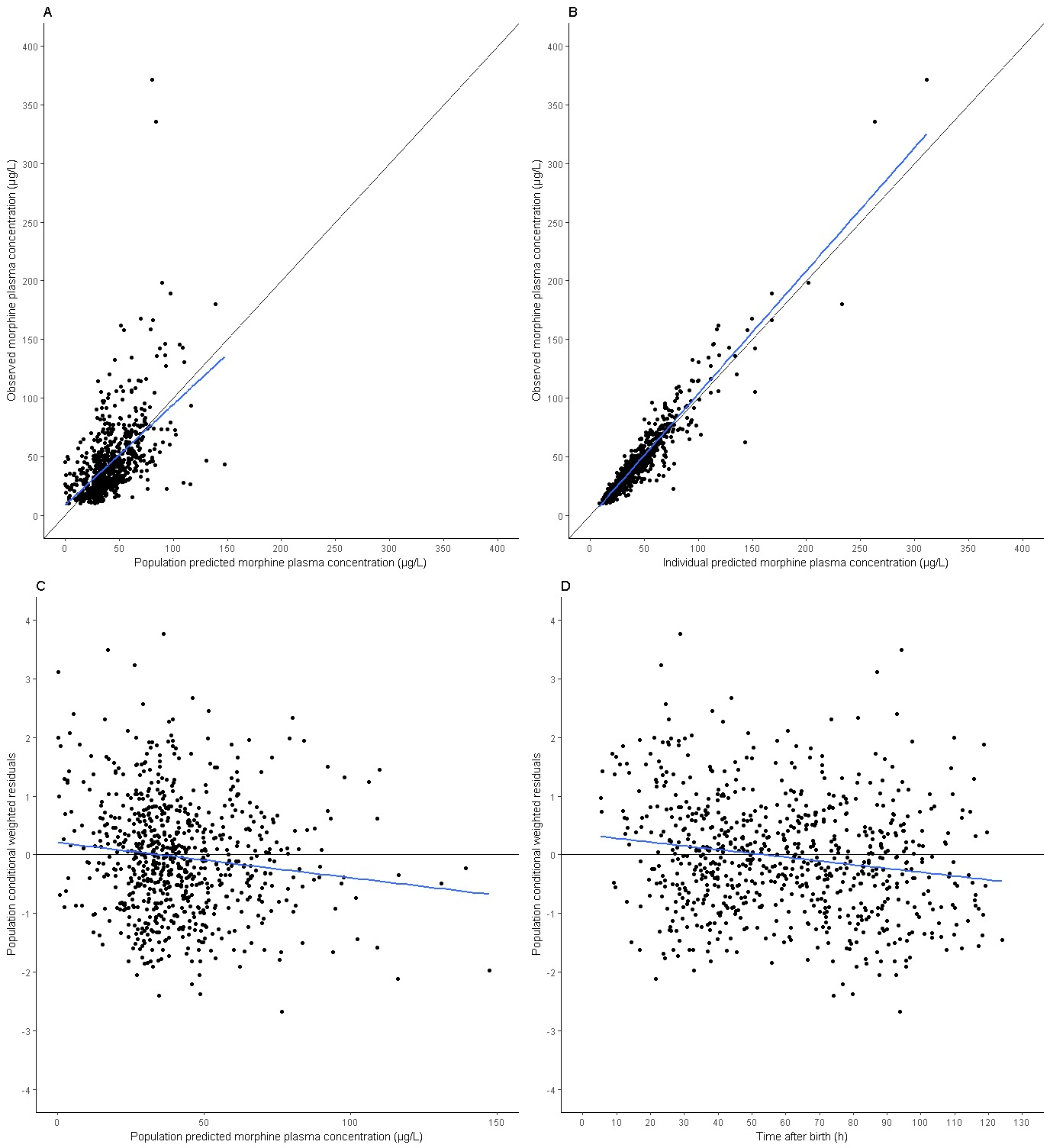

Supplement: S2 Fig — A = observed vs population predicted plasma concentrations; B = observed vs individual predicted plasma concentrations; C = population conditional weighted residuals vs population predicted plasma concentrations; D = population conditional weighted residuals vs time after birth; solid line indicates the linear regression line. (TIF) [file pone.0211910.s002.tif]

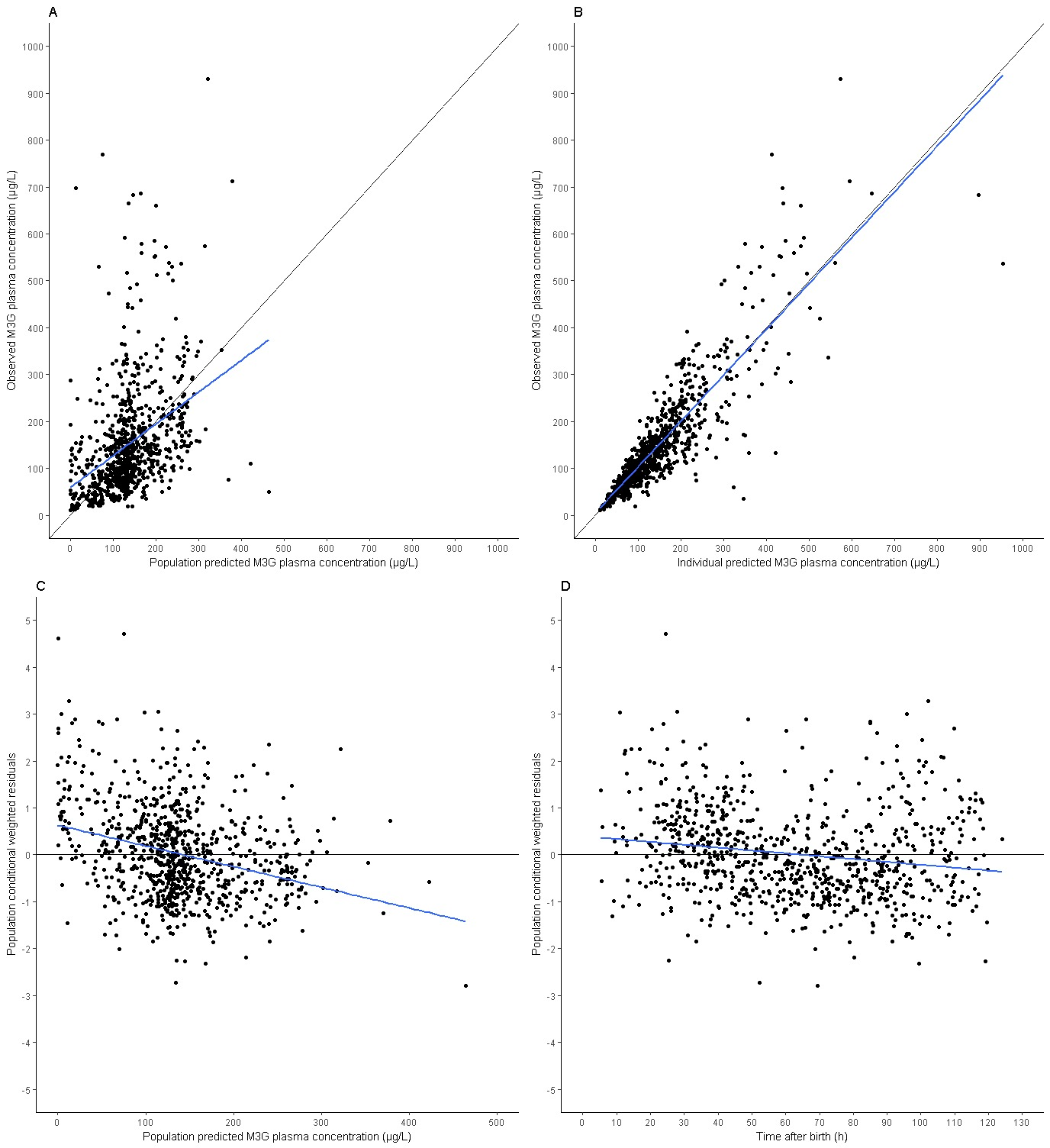

Supplement: S3 Fig — A = observed vs population predicted plasma concentrations; B = observed vs individual predicted plasma concentrations; C = population conditional weighted residuals vs population predicted plasma concentrations; D = population conditional weighted residuals vs time after birth; M3G = morphine-3-glucuronide; solid line indicates the linear regression line. (TIF) [file pone.0211910.s003.tif]

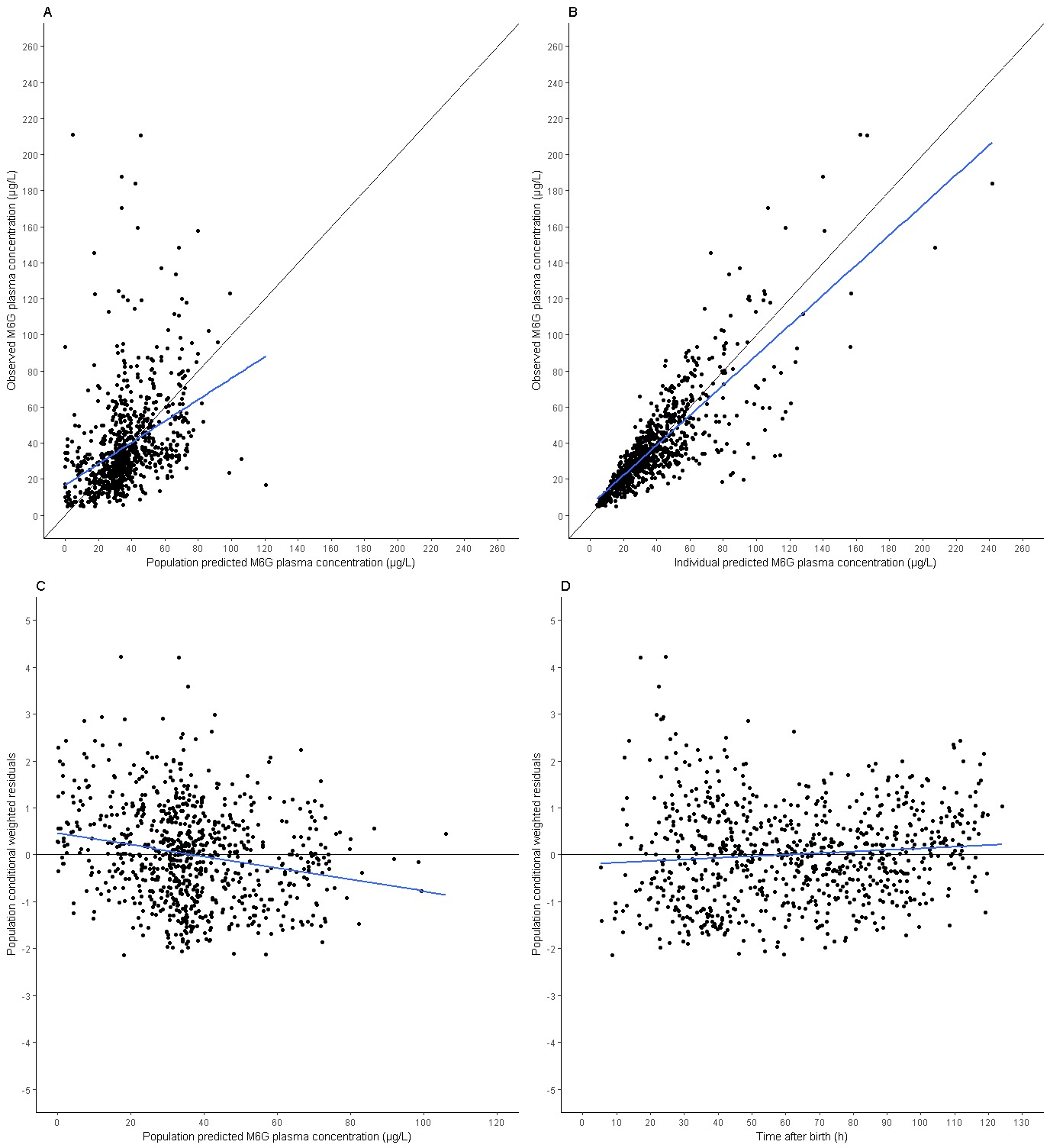

Supplement: S4 Fig — A = observed vs population predicted plasma concentrations; B = observed vs individual predicted plasma concentrations; C = population conditional weighted residuals vs population predicted plasma concentrations; D = population conditional weighted residuals vs time after birth; M6G = morphine-6-glucuronide; solid line indicates the linear regression line. (TIF) [file pone.0211910.s004.tif]

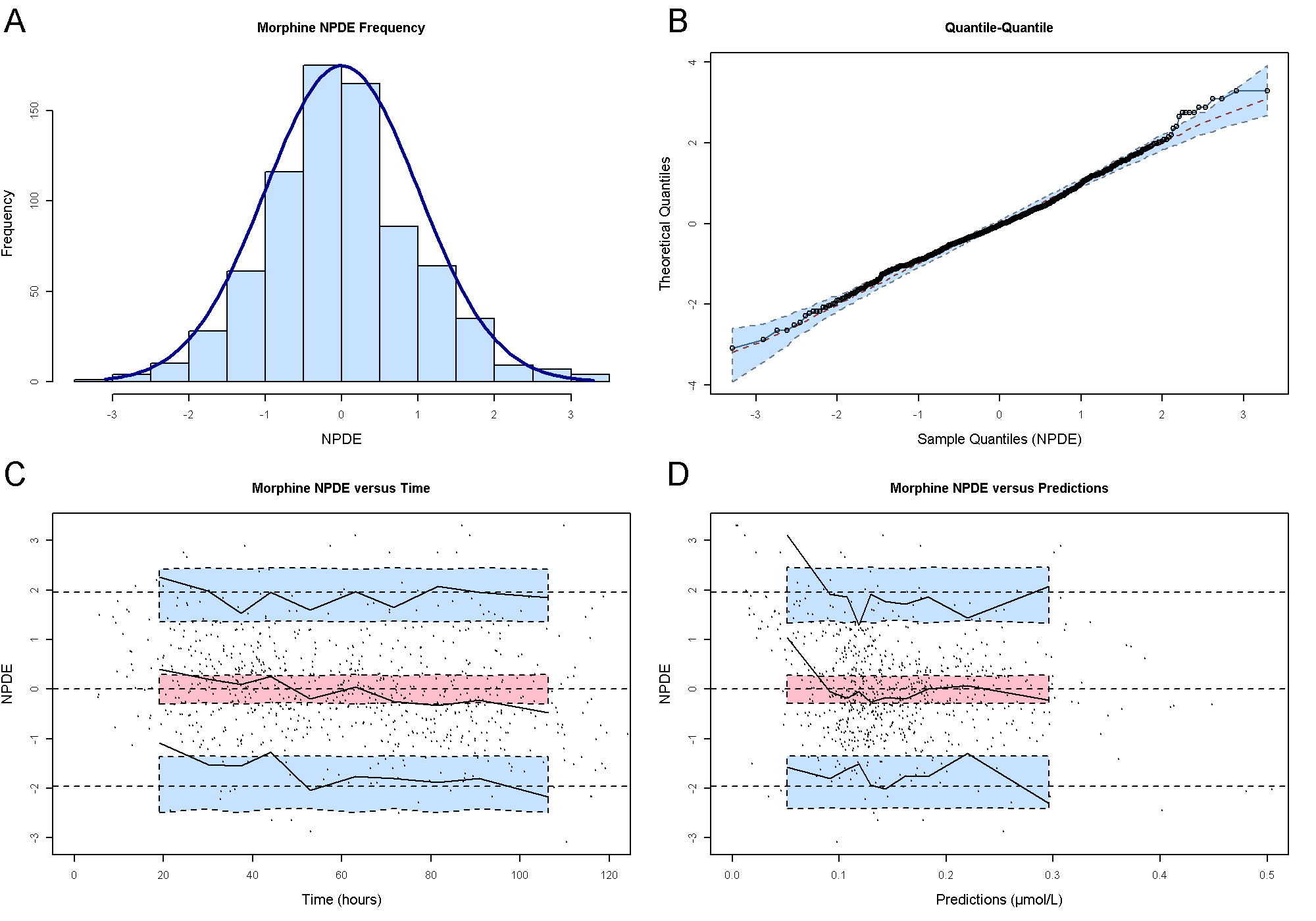

Supplement: S5 Fig — A = kernel density plot of NPDE with a normal, Gaussian distribution overlaid for comparative purposes; B = Q-Q plot of theoretical quantiles vs sample quantiles; C = NPDE vs Time; D = NPDE vs predicted plasma concentrations; solid lines in figures C and D represent the observed median, 5th and 95th percentiles, red box represent the predicted 90% confidence interval around the median, blue boxes represent the predicted 90% confidence intervals around the 5th and 95th percentiles. (TIF) [file pone.0211910.s005.tif]

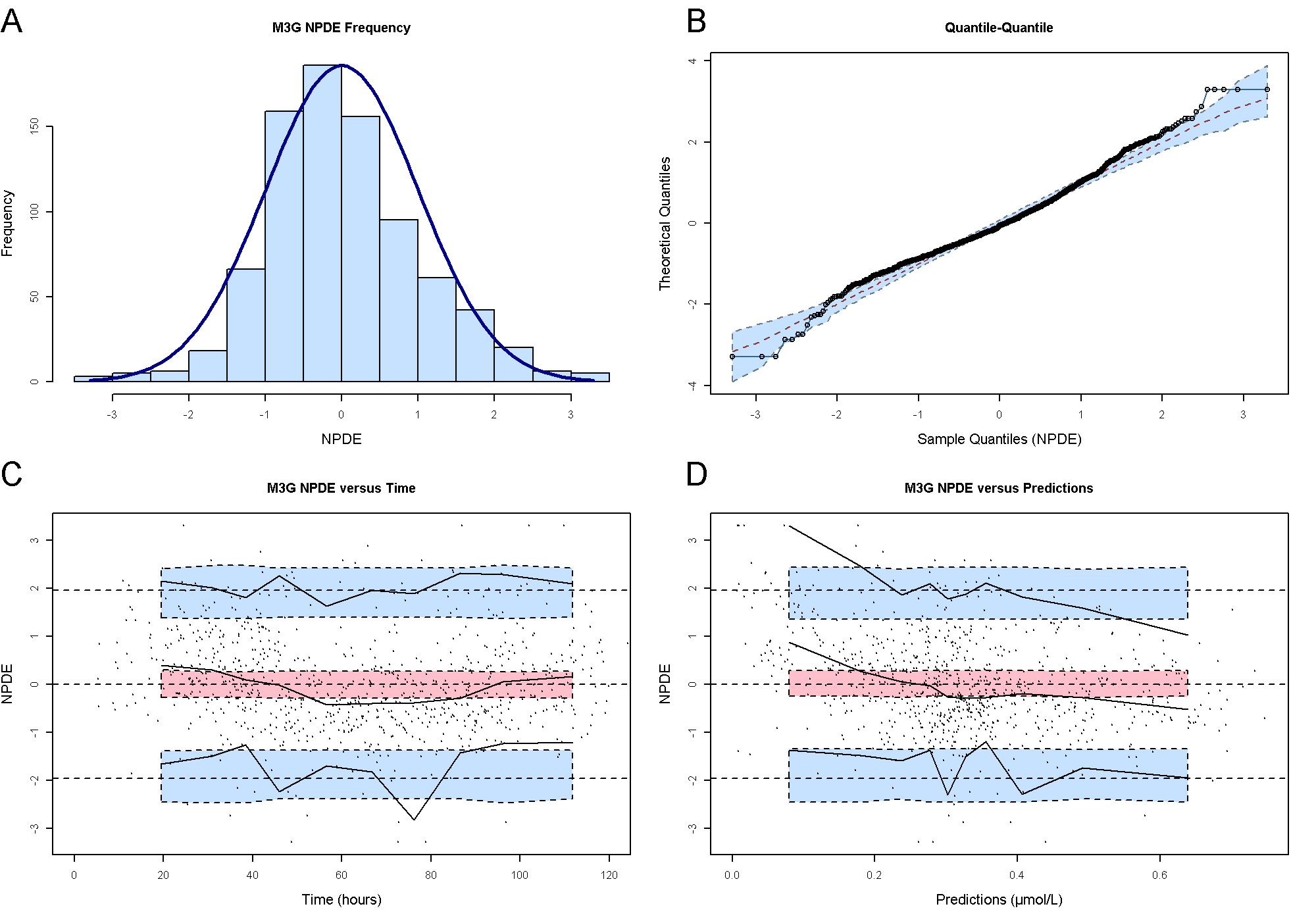

Supplement: S6 Fig — A = kernel density plot of NPDE with a normal, Gaussian distribution overlaid for comparative purposes; B = Q-Q plot of theoretical quantiles vs sample quantiles; C = NPDE vs Time; D = NPDE vs predicted plasma concentrations; M3G = morphine-3-glucuronide; solid lines in figures C and D represent the observed median, 5th and 95th percentiles, red box represent the predicted 90% confidence interval around the median, blue boxes represent the predicted 90% confidence intervals around the 5th and 95th percentiles. (TIF) [file pone.0211910.s006.tif]

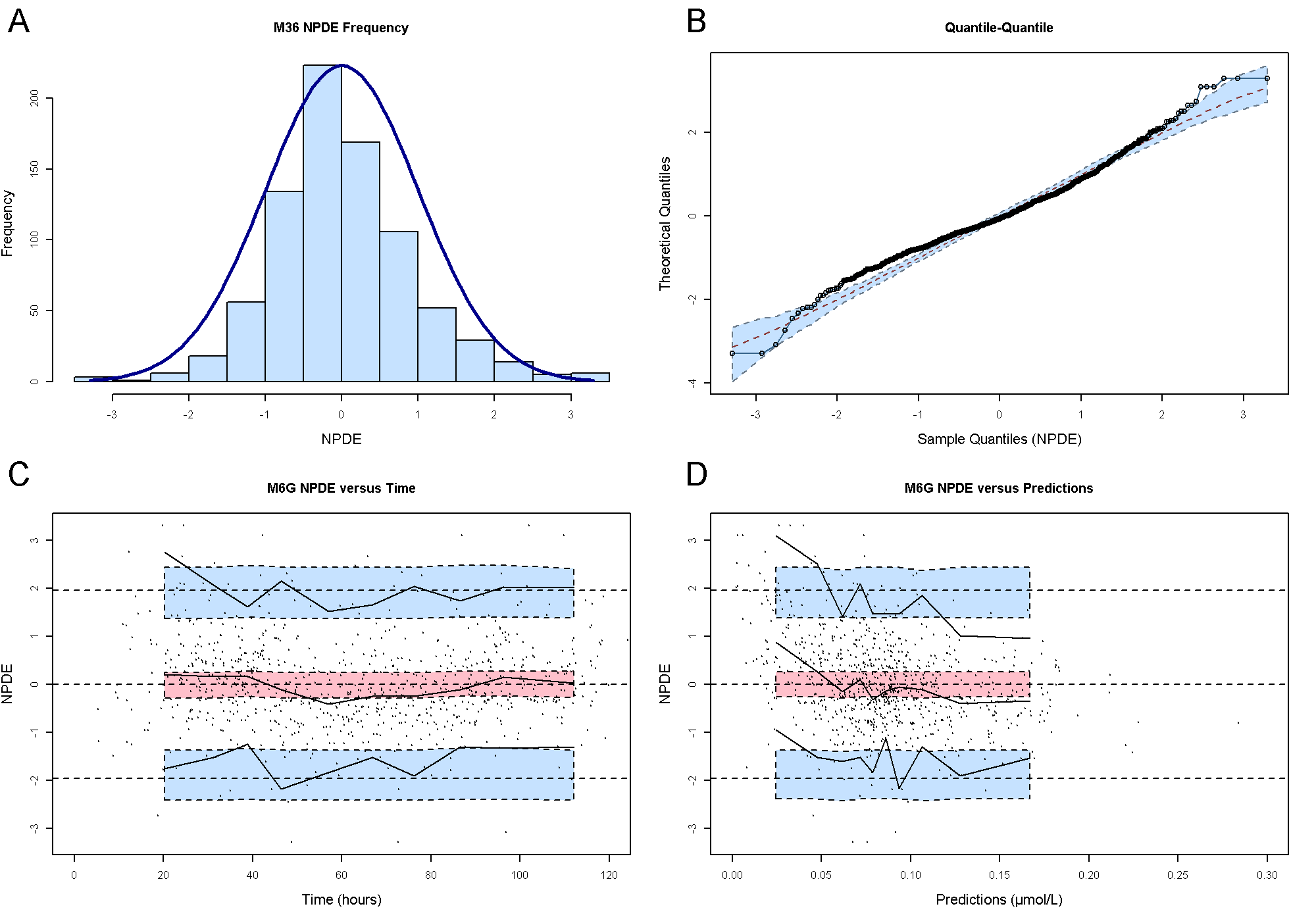

Supplement: S7 Fig — A = kernel density plot of NPDE with a normal, Gaussian distribution overlaid for comparative purposes; B = Q-Q plot of theoretical quantiles vs sample quantiles; C = NPDE vs Time; D = NPDE vs predicted plasma concentrations; M6G = morphine-6-glucuronide; solid lines in figures C and D represent the observed median, 5th and 95th percentiles, red box represent the predicted 90% confidence interval around the median, blue boxes represent the predicted 90% confidence intervals around the 5th and 95th percentiles. (TIF) [file pone.0211910.s007.tif]

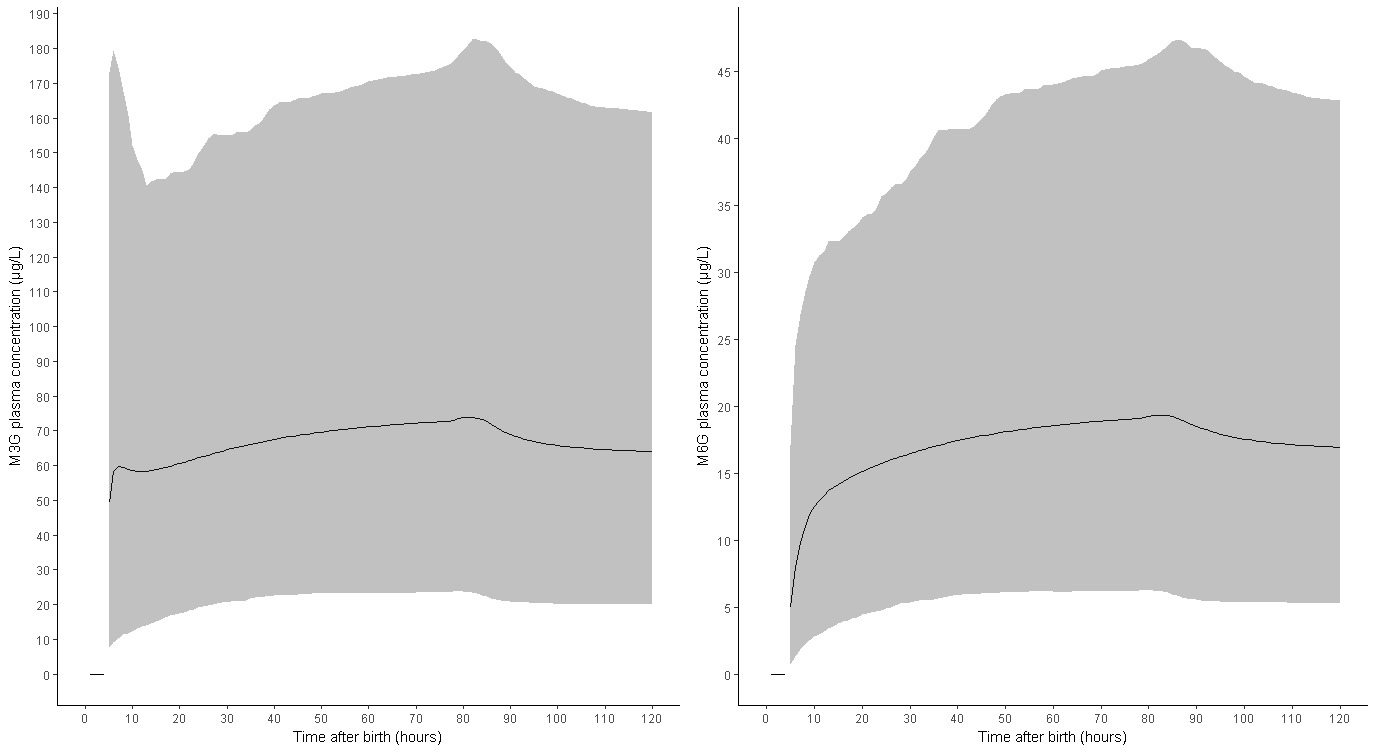

Supplement: S8 Fig — simulated plasma concentration time profiles for M3G (left) and M6G (right) of the proposed morphine dosing regimen of 5 μg/kg/h after loading dose of 50 μg/kg. Solid line indicates the mean plasma concentration; gray area represents the 95% prediction interval. M3G = morphine-3-glucuronde, M6G = morphine-6-glucuronide. (TIF) [file pone.0211910.s008.tif]

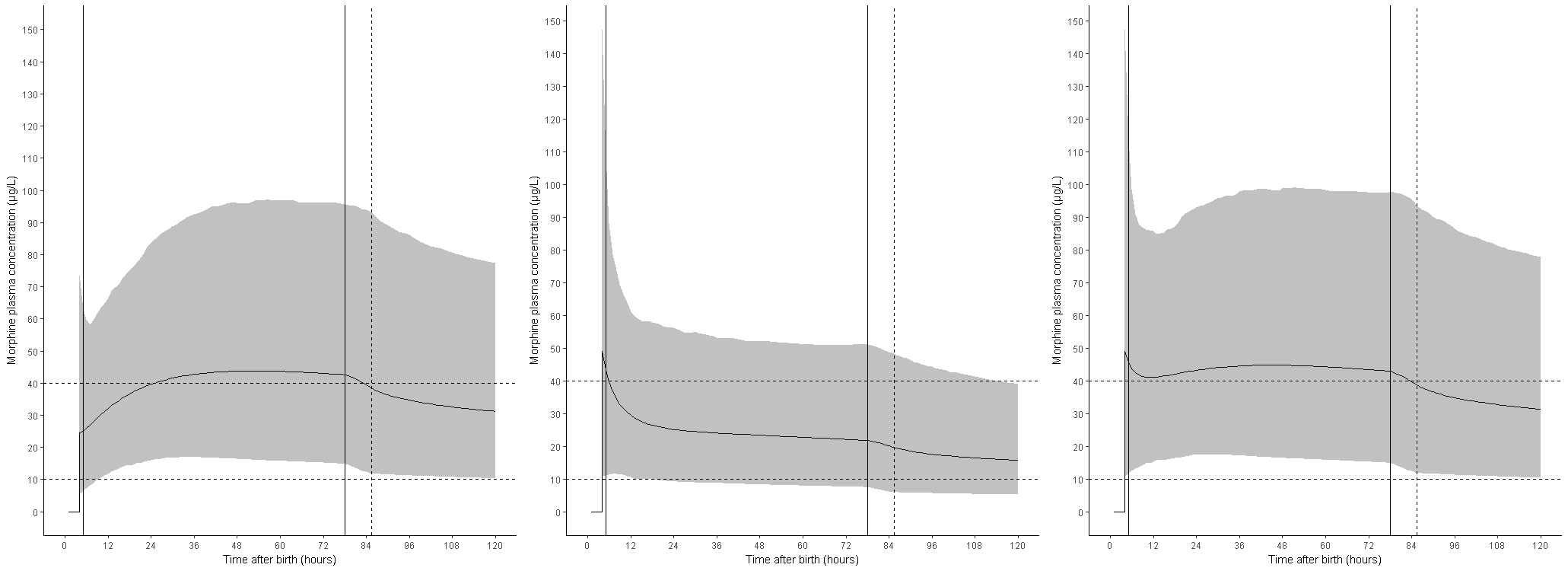

Supplement: S9 Fig — Simulated morphine plasma concentrations of the dosing regimens of 10 μg/kg/h after loading dose of 50 μg/kg (left), 5 μg/kg/h after loading dose of 100 μg/kg (center) and 10 μg/kg/h after loading dose of 100 μg/kg (right). Solid line indicates the mean morphine plasma concentration; gray area represents the 95% prediction interval. Dotted horizontal lines indicate the proposed therapeutic window of 10–40 μg/L. Solid vertical lines indicate the start and end of TH (33.5°C) simulated between 5h and 77h after birth; dashed vertical line indicates the return to normothermia (36.5°C) with rewarming simulated at 0.4°C/h; TH = therapeutic hypothermia. (TIF) [file pone.0211910.s009.tif]
